# UNC-33L partially rescues life span and locomotion defects in *unc-33* mutants but fails to rescue dauer formation defects.

**DOI:** 10.17912/micropub.biology.000515

**Published:** 2022-01-24

**Authors:** Melissa E Lopez, Arianna M Vacio, Jason Cantu, Andrea Holgado

**Affiliations:** 1 Department of Biological Sciences, St. Edward's University, Austin, TX

## Abstract

Herein, we tested the ability of UNC-33L to rescue dauer formation, lifespan, and locomotion defects of *unc-33(mn407) *mutants. Results show that the presence of UNC-33L does not rescue the defective dauer phenotype in *unc-33(mn407)* mutants. However, UNC-33L significantly rescued premature death and uncoordinated locomotion in young *unc-33(mn407)* adults. The degree of UNC-33L-mediated rescue was less noticeable as the nematodes aged, denoting that both age and the presence of UNC-33L interact in the production of the phenotypes.

**Figure 1. Effect of UNC-33L in dauer formation, lifespan, and locomotion of unc-33 mutants:**
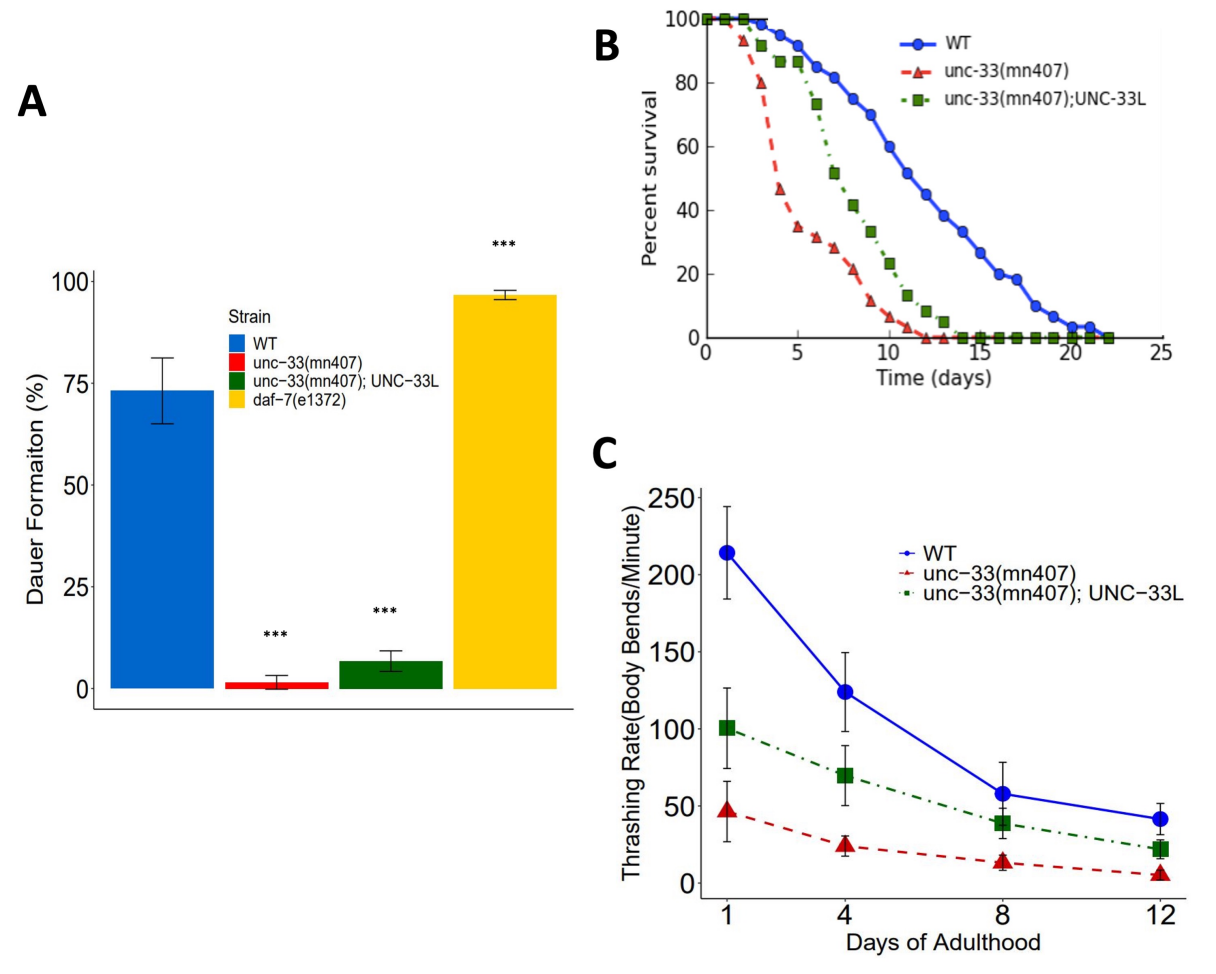
(A) Analysis of resistance to SDS in *C. elegans* dauers was measured as percent survival after treatment with 1% SDS. Plotted is the average of three replicas +/- the standard deviation, Statistical analysis was performed using one-way ANOVA and Tukey Post Hoc for pairwise comparisons. *** *p-value* ≦0.001. (B) Lifespan of *C. elegans* adult hermaphrodites was quantified during 22 days of adulthood. Plotted is the mean of a population of 60 animals obtained as three biological replicas of 20 nematodes per genotype. Data were analyzed using Oasis (Online Application for Survival Analysis) (Yang *et al.* 2011). (C) Thrashing rates in liquid media were quantified for nematodes 1, 4, 8, and 12 days of adulthood. The averages of thrashing rates are plotted +/- standard deviations. Statistical analyses were performed using two-way ANOVA. *p-values* ≦0.001 were obtained in comparisons among strains, among time, and a significant interaction effect (*p-value* ≦0.001) was detected between strain and time.

## Description

*C. elegans* have a short life cycle that consists of a larva molting into a reproducing adult by passing through 4 larval stages (L1, L2, L3, and L4). Under favorable conditions, the nematode will go through all 4 larval stages (Wolkow and Hall 2015). On the other hand, if conditions become unfavorable, the nematode will shift into dauer formation right after the L2 phase (L2d). During this alternative life cycle, *C. elegans* become stress-resistant, developmentally arrested, and long-lived (Albert and Riddle 1983). In addition, they undergo morphological remodeling that allows them to adapt to harsh conditions (Cassandra and Russell 1975). Dauers develop a thickened cuticle, undergo body constriction, and cell compaction. The thickened cuticle inhibits environmental toxins, such as detergent from affecting internal organs (Wolkow and Hall 2015). Once conditions become favorable, the nematode will exit out of the dauer phase and continue on to become a reproducing adult.

Although wild-type (WT) nematodes can enter this dauer phase, *unc-33* mutants are unable to enter this alternative life cycle, and the reasoning behind this is unclear. The *C. elegans* UNC-33 protein is the ortholog of human microtubule-binding protein CRMP2 (Tsuboi *et al.* 2005). UNC-33/CRMP2 plays a role in microtubule organization and axon-dendrite sorting to establish an organism’s nervous system (Maniar *et al*. 2011). In the absence of UNC-33 polypeptides, nematodes present with uncoordinated locomotion and defects in axonal elongation (Li *et al.* 1992). In humans, studies have found CRMP2 defects in patients with Alzheimer’s and other age-related neurodegenerative diseases (Ban *et al.* 2013; Arey and Murphy, 2017).

Aging is a biological phenomenon that occurs in all life forms due to the decline and deterioration of cells and organs. Aging cells and organs progressively accumulate dysfunctional cellular components, are subject to oxidative damage, and have a decline in protein turnover rates and housekeeping mechanisms (Kurz *et al.* 2007; Rajawat and Bossis 2008). Evidence collected from the study of aging suggests that the continuous removal of dysfunctional organelles and the synthesis of new ones allow for a delay in the aging process, gleaning that there are mechanisms extending longevity and inhibiting aging. Further exploring the aging mechanisms is the objective of this study.

To analyze the effects of the *unc-33* gene in *C. elegans* further, we investigated the role of UNC-33L in dauer development, lifespan, and age-dependent locomotion in *unc-33(mn407)* mutants. UNC-33 codes for three alternatively spliced isoforms UNC-33L (Large), UNC-33M (Medium), and UNC-33S (Small) with each isoform varying at the N terminus (Maniar *et al.* 2011). *unc-33(mn407)* mutants contain a 500 bp deletion in the *unc-33* ORF and produce none of the alternatively spliced isoforms of UNC-33 (Tsuboi *et al.* 2005 and Maniar *et al*. 2011). In previous studies, Maniar and colleagues found that UNC-33L was able to fully rescue uncoordinated locomotion, egg-laying defects, and mislocalization of RAB-3::mCherry and SAD-1::GFP in *unc-33* *(mn407)* mutants, suggesting that UNC-33L is sufficient to restore neuronal activity to WT levels in the *unc-33* mutant background (Maniar *et al.* 2011). To this end, we hypothesize that the introduction of UNC-33L in an *unc-33* null mutantwill result in a rescue of the dauer defective phenotype, lifespan, and age-dependent locomotion.

To test the rescue of the dauer defective phenotype, we treated nematodes with 1% SDS and assessed dauer formation. Analysis of our results show that *unc-33(mn407)*;UNC-33L transgenic nematodes were unable to produce dauers (6.74% +/- 0.025%), a phenotype characteristic of *unc-33(mn407)* mutants (3.23% +/- 0.026%). Meanwhile, dauer formation was scored as 96.68% +/- 0.011% in *daf-7(e1372)* mutants and 73.14% +/- 0.081% in WT nematodes (Figure 1A). Examinations of pairwise comparisons via the Tukey post-hoc resulted in no statistically significantly different results between *unc-33(mn407)*; UNC-33L transgenics and *unc-33(mn407)* mutants (*p-value=* 4.98E-01*)*. However, pairwise comparisons between the remaining strains resulted in statistically significantly different results as follows: *unc-33(mn407);* UNC-33L vs WT *p-value* ≦0.0001; *daf-7(e1372)* vs *unc-33(mn407)* *p-value* ≦0.0001; *unc-33(mn407);* UNC-33L vs *daf-7(e1372) p-value* ≦0.0001, WT vs *unc-33(mn407)* *p-value* ≦0.0001;and WT vs *daf-7(e1372) p-value* ≦0.0001. These results suggest that the addition of UNC-33L was not sufficient in rescuing the defect in dauer formation of the *unc-33(mn407)* mutants as scored by the 1% SDS protocol.

To test the effects of UNC-33L rescue in lifespan, we quantified the longevity in *unc-33* mutants and found a partial rescue denoted by a *p-value* ≦0.0001 in the pairwise comparison of *unc-33(mn407)* vs *unc-33(mn407);* UNC-33L transgenics using a Kaplan-Meier test. Analysis of WT vs *unc-33(mn407*); UNC-33L pairwise comparison had a *p-value* ≦0.0001. The Kaplan-Meier estimates of 50 percent mortality were 12 days of adulthood for WT, 8 days of adulthood for *unc-33(mn407)*; UNC-33L transgenics, and 5 days of adulthood for *unc-33(mn407)* mutants. Additionally, examinations of lifespan assays showed that animals expressing UNC-33L had death rates similar to WT during days 1-5 of adulthood. Following day 5 of adulthood, *unc-33(mn407)*; UNC-33L nematodes showed an increase in death rates, resulting in a phenotype intermediate between WT animals and *unc-33(mn407)* mutants (Figure 1B).

Lastly, in testing whether locomotion defects would be rescued in the presence of UNC-33L, we quantified thrashing rates in *unc-33* mutants and found a significant difference in thrashing rates between *unc-33(mn407)* vs *unc-33(mn407*); UNC-33L transgenics (Figure 1C). Analysis of the data using two-way ANOVA showed an interaction effect between strains and age, demonstrating that the degree of the rescue of thrashing is dependent on the age of the nematodes (Figure 1C).

Together, lifespan and locomotion studies suggest that expression of UNC-33L alone has a greater effect on survivability and motility earlier in adulthood. However, this positive effect declines as the animals’ age, resulting in the partial rescue of lifespan and locomotion defects.

## Methods

Synchronization of nematodes:

To synchronize nematodes, adult worms from each strain were floated in DI water, transferred to 15 mL conical tubes, and centrifuged for 1 minute at 3757 x g at 20 ºC. After centrifugation, the supernatant was discarded, and 7 mL of alkaline bleach plus 7 mL of DI water were added to the pellet. The tube containing the pelleted worms and diluted alkaline bleach was incubated for 7 minutes on a rocker at room temperature. After incubation, the worm suspension was centrifuged for 5 minutes at 3757 x g at 20 ºC, and the supernatant was discarded. Next, the pellets were washed using 10 mL of M9 buffer and centrifuged for 1 minute at 9221 x g at 20ºC after each wash. A total of three washes were performed. After the final centrifugation, the supernatant was discarded, and the pellets containing eggs were pipetted onto seeded plates.

Lifespan Assay:

The longevity of nematodes was quantified by examining 20 synchronized young adults per strain and assessing their survival every 24 hours until death. Synchronized populations of worms were cultivated on NGM agar plates containing OP50 *E. coli* bacteria at 20 ºC. Once they’ve reached adulthood, 20 nematodes per strain were transferred onto a new plate and recorded as day 1 of adulthood. The survival of nematodes was monitored daily by transferring living animals to a new seeded plate with OP50 *E. coli* until all remaining animals died. Survival was recorded as the percent ratio of living to dead nematodes.

Locomotion Assay:

The locomotion of the nematodes was quantified by examining 20 adults per strain and assessing their body thrashes in liquid at various ages of their adult life (1, 4, 8, and 12 days of adulthood). Synchronized populations of worms were cultivated on NGM agar plates containing OP50 *E. coli* bacteria at 20 ºC. Once they’ve reached the specified adult age, individual nematodes were picked and transferred to wells containing 1 mL of DI water. Once transferred, each nematode was allowed to acclimate for 10-15 seconds before evaluating thrashing rates. Locomotion is quantified by counting the thrashes in 30 seconds, and the thrashing rate was expressed as body bends per minute.

SDS Protocol:

To assess dauer formation, we examined 200 starved worms per strain, per trial. Animals were starved for 5 days at 20 ºC, with the exception of *daf-7(e1372)* which were kept at 25 ºC for 5 days. After the 5-day starvation period was reached, animals were resuspended with 1 mL DI water and transferred into 1.5 mL tubes. Worms were centrifuged for 1 minute at 9221 x g and pellet worms were treated with 1 mL of 1% SDS for 20 min at room temperature on an orbital shaker. After the 20 minute incubation, worms were centrifuged for 1 minute at 9221 x g and the supernatant was removed. Pelleted worms were washed 3 times using 1 mL of M9 buffer and centrifuged for 1 minute at 9221 x g at 20ºC after each wash. After the last wash, 100uL of pellet worms were transferred onto an NGM plate with no bacteria and the percent survival was quantified. Alive animals were picked one by one and then burned to ensure adequate scoring.

Statistical Analysis:

All data analysis was performed using R. For dauer formation, hypothesis testing was evaluated using one-way ANOVA after determining normality via the Shapiro-Wilks test and homogeneity of variance via the Levene test. A Post-Hoc Tukey’s test was used for pairwise comparisons. Lifespan assays were analyzed via Kaplan-Meier Survival Analysis using Oasis programming (Yang *et al*. 2011). The locomotion data was interpreted using two-way ANOVA after determining normality via the Shapiro-Wilks test and homogeneity of variance via the Levene test.

## Reagents

N2 (WT), SP1382 *unc-33(mn407)*, and CB1372 *daf-7(e1372)* nematodes were obtained from the *Caenorhabditis* Genetics Center.

Strain CX13244 kyIs445 [PVD::mcherry::RAB-3 + PVD::SAD-1::GFP]; *unc-33(mn407); kyEx3861* [tag-168::UNC-33L (20 ng/μ*l), unc-122::DsRed (20 ng/*μl)], was obtained from Cori Bargmann’s Lab (Maniar *et al.*. 2011). Strain CX13244 was created by microinjecting a plasmid driving the expression of an UNC-33L cDNA under the regulation of the pan-neuronal *tag-168* promoter.
